# Federated learning for diagnosis of age-related macular degeneration

**DOI:** 10.3389/fmed.2023.1259017

**Published:** 2023-10-12

**Authors:** Sina Gholami, Jennifer I. Lim, Theodore Leng, Sally Shin Yee Ong, Atalie Carina Thompson, Minhaj Nur Alam

**Affiliations:** ^1^Department of Electrical Engineering, University of North Carolina at Charlotte, Charlotte, NC, United States; ^2^Department of Ophthalmology and Visual Science, University of Illinois at Chicago, Chicago, IL, United States; ^3^Department of Ophthalmology, School of Medicine, Stanford University, Stanford, CA, United States; ^4^Department of Surgical Ophthalmology, Atrium-Health Wake Forest Baptist, Winston-Salem, NC, United States

**Keywords:** FL, deep learning, optical coherence tomography, residual network, vision transformers, AMD, domain adaptation, adaptive personalization FL

## Abstract

This paper presents a federated learning (FL) approach to train deep learning models for classifying age-related macular degeneration (AMD) using optical coherence tomography image data. We employ the use of residual network and vision transformer encoders for the normal vs. AMD binary classification, integrating four unique domain adaptation techniques to address domain shift issues caused by heterogeneous data distribution in different institutions. Experimental results indicate that FL strategies can achieve competitive performance similar to centralized models even though each local model has access to a portion of the training data. Notably, the Adaptive Personalization FL strategy stood out in our FL evaluations, consistently delivering high performance across all tests due to its additional local model. Furthermore, the study provides valuable insights into the efficacy of simpler architectures in image classification tasks, particularly in scenarios where data privacy and decentralization are critical using both encoders. It suggests future exploration into deeper models and other FL strategies for a more nuanced understanding of these models' performance. Data and code are available at https://github.com/QIAIUNCC/FL_UNCC_QIAI.

## 1. Introduction

Age-related macular degeneration (AMD) is a common eye condition and a leading cause of vision loss among people aged 50 and older (1). AMD causes damage to the macula, the part of the eye that provides sharp, central vision, which is located near the retina's center. As a result, everyday activities such as reading and driving may be difficult to perform. In order to prevent severe vision impairment and preserve vision, detection of AMD in its early stages is crucial to implementing appropriate treatments, such as medications or procedures. Artificial intelligence (AI) can play a pivotal role in the preliminary identification and classification of AMD (2–8). Its proficiency in discerning the disparate stages of both wet and dry AMD results in substantial enhancement of the prognosis of treatment outcomes. Deep learning (DL) models significantly refine the precision and accuracy of AMD diagnosis, capable of detecting subtle ocular changes that might elude human scrutiny (3). The remarkable capacity of AI for rapid analysis of imaging data facilitates more expeditious and efficient diagnosis, a critical factor in timely disease management (9).

The broad applications of AI include large-scale AMD screening within populations, a critical feature, particularly in areas where accessibility to ophthalmologists is restricted (10). Beyond these clinical uses, AI's potential to discern patterns and correlations in expansive datasets could yield innovative perspectives into the origins and evolution of AMD, potentially influencing future research trajectories (11).

AI models employed in AMD diagnosis predominantly utilize centralized learning. This traditional method accumulates data from diverse sources, collating them in a centralized server or location for the training of a machine learning (ML) model (12). Adherence to data protection regulations such as the Health Insurance Portability and Accountability Act (HIPAA) is paramount in healthcare environments (13). Thus, this approach encounters hurdles due to data privacy and security concerns in the medical sphere.

The introduction of federated learning (FL) allows model training without the dissemination of raw patient data, thereby circumventing privacy issues as data remains local. The possibilities proffered by FL involve enhancing diagnostic accuracy, prediction capability, and personalized treatment within ophthalmology, whilst harnessing large, diverse datasets from multiple institutions. However, for successful FL integration, it is necessary to address challenges linked with data heterogeneity, along with assuring the reliability and security of the learning process.

FL has shown significant potential in healthcare for addressing challenges related to data security and collaboration. Dayan et al. (14) showcased the effectiveness of FL in predicting the oxygen needs of COVID-19 patients across 20 global institutes, underlining its potential for swift data science collaboration in healthcare without the need for direct data sharing. In the realm of ophthalmology, a study by Lu et al. (15) found that 57% of models trained on individual institutional data were surpassed by FL models, emphasizing the advantage of FL in multi-institutional learning, especially beneficial for smaller institutions with limited resources. Sadilek et al. (16) highlighted the advancements in FL that ensure robust privacy protections while integrating differential privacy into clinical research. Another study focused on retinopathy of prematurity (ROP), where a DL model trained via FL with data from 5,245 patients across seven institutions identified diagnostic disparities and suggested standardization potential in clinical diagnoses (17). Furthermore, Lu et al. (15) demonstrated that FL-trained models for ROP diagnosis exhibited comparable performance to centralized models. Investigating diabetic retinopathy leveraged FL's potential to develop more generalized models by utilizing diverse datasets without compromising data privacy (18). Lastly, a comprehensive review by Nguyen et al. (19) emphasized the transformative potential of DL in ocular imaging, with FL providing an effective solution to data security concerns.

Inconsistencies in optical coherence tomography (OCT) image acquisition parameters and scanning protocols can induce variations in image quality (20). Clinical and technical hurdles including differing standards and regulations among various Institutional Review Boards (IRBs), and limited training datasets for rare diseases can exacerbate the complexities of constructing and implementing DL techniques (21). Such variations can impact the competence and generalizability of DL models (22).

The domain shift problem also poses a significant challenge in the context of FL (23, 24). Domain shift arises when there is a substantial difference in data distributions across various local devices or nodes, also termed as clients, within the FL system. The non-identically distributed nature of decentralized data, a key characteristic of FL, can potentially compromise model learning performance (25). Rectifying this issue necessitates strategic and robust methodologies.

In the research of Li et al. (25), domain adaptation (DA) techniques are outlined for optimizing learning algorithms irrespective of disparities in data distribution. Employing domain-invariant features or transfer learning methodologies, these techniques endeavor to lessen the impact of varied data distributions. Additionally, data augmentation can be leveraged to artificially enhance data representation, thereby diminishing the effects of domain shift (26). Other methods can also be utilized to counter this challenge, encompassing client selection and sampling strategies, model aggregation procedures, proactive domain exploration (27), and FL personalization (28). By effectively tackling domain shifts, FL can bolster the model's generalization capacity and augment performance across disparate domains.

The purpose of this manuscript is to delineate the practicality of employing DA FL in the diagnosis of AMD. There is potential for domain shifts due to variations in protocols and OCT machines used for retinal imaging in the collaborative development of a classification model across institutions. Using data from three distinct datasets, this study examines various FL strategies to address this issue for AMD retinal OCT binary classification, utilizing an open-source FL Python library. The performance of these FL strategies was compared with a baseline centralized approach, emphasizing the potential benefits of employing multiple FL techniques to counteract the domain shift. However, this research did not delve into the security aspects of the FL framework, and all the involved entities, including the server and the FL node, were reliable and did not distribute distorted data or behave maliciously.

## 2. Methods

### 2.1. Data

We leveraged OCT data derived from three distinct research datasets for our study: Kermany et al. (29), Srinivasan et al. (30), Li et al. (31), hereinafter referred to as DS1, DS2, and DS3. The utilization of these distinct datasets facilitated the simulation of three disparate institutions (FL nodes) intent on training a DL model for binary image classification (Normal vs. AMD). Hence, each node is allocated its own training, validation, and testing set.

DS1 encompasses a total of 84,484 OCT retinal (Spectralis OCT, Heidelberg Engineering, Germany) images from 3,919 patients which are classified into four categories: Normal, Choroidal Neovascularization, Diabetic Macular Edema (DME), and Drusen. These images are compartmentalized into three separate folders: training, validation, and testing. However, it was observed that some images were duplicated across the validation and testing folders as well as the training folder. To eliminate redundancy, we amalgamated the validation and testing folders and compared each image with those in the training set using the mean square error (MSE) technique. An MSE score of zero signified the presence of identical images, leading to the identification of 8,520 duplicates within the dataset. These issues originated from 34 images that were marked as both normal and diseased retina. We discarded these images and exclusively used Normal and Drusen (ADM) retinal images for this binary classification task. In the end, around 3% of the patient samples were chosen as the test set, which contained varying numbers of scans per patient.

DS2 contains retinal images from 45 subjects, which includes 15 individuals each from the categories of Normal retinas, AMD, and DME. For training purposes, we used data from the first 11 Normal and AMD patients. Data from the 12th subjects with Normal and AMD retinas were designated for validation, while the remaining data served to test the model. All the OCT volumes were acquired in IRB-approved protocols using Heidelberg Engineering Spectralis SD-OCT (30).

DS3 encompasses OCT images from 500 subjects, captured under two distinct fields of view: 3 and 6-mm. A single 3-mm file consists of 304 scans from an individual patient, whereas a 6-mm file holds 400 scans. Subsequently, we isolated the images of Normal and AMD retinas. Recognizing the limited significance of peripheral retinal sections in classification, our attention was centered on the fovea images, specifically image numbers 100–180 for the 3-mm scans and 160–240 for the 6-mm scans. All OCT images were captured using a spectral-domain OCT system with a center wavelength of 840 nm (RTVue-XR, Optovue, CA) (31).

**Figure 1:**
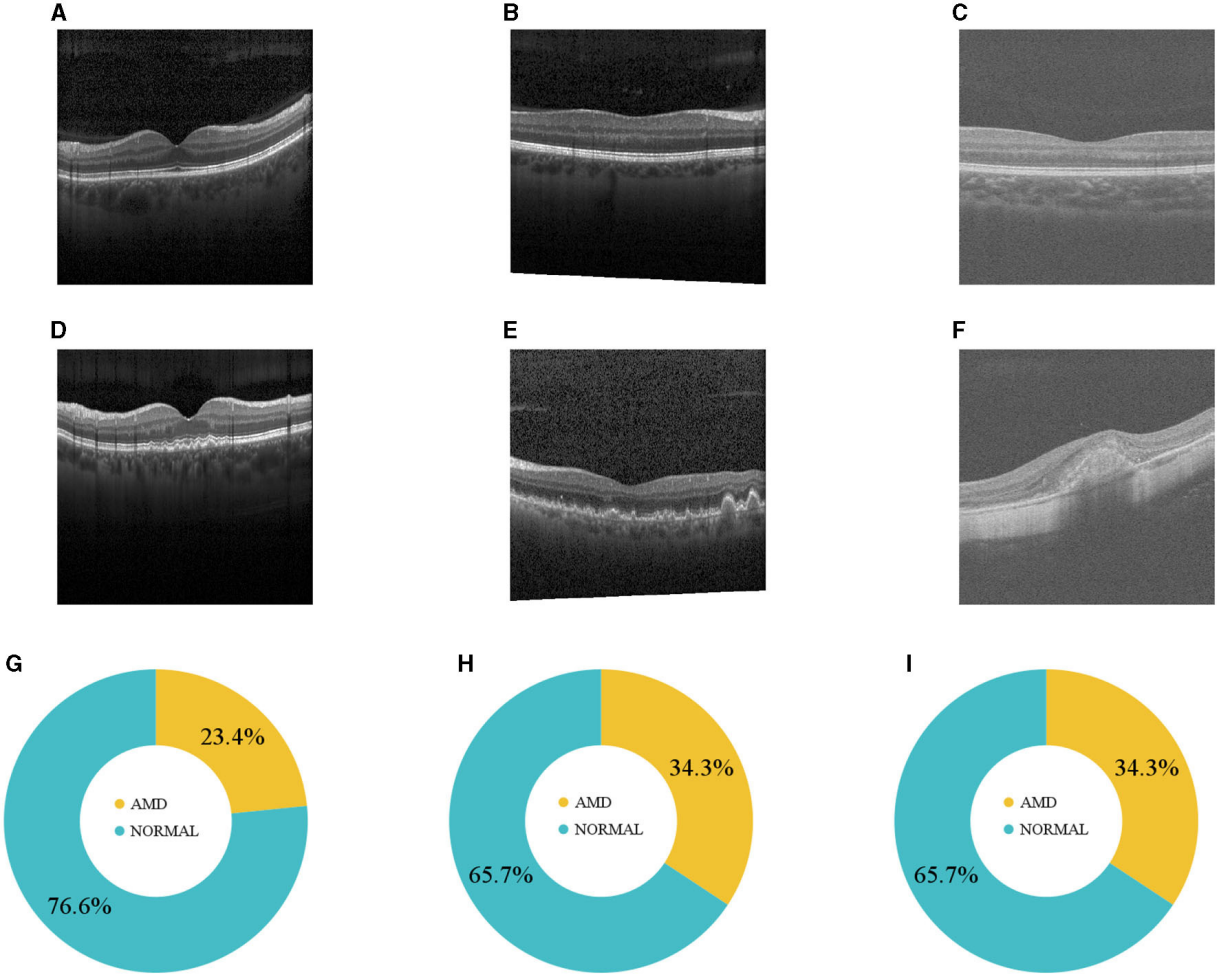
**(A–C)** Normal and **(D–F)** AMD sample OCTs from the three datasets. **(G–I)** Illustrate how DS1, DS2, and DS3 are distributed.

The distribution of the data across the three datasets is visually represented in Figure 1 and tabulated in Table 1. The size of DS2 is relatively smaller compared to other datasets. This mirrors the common real-world scenario where certain participants contributing to training have limited data. The datasets for training, validation, and testing have been resized to a resolution of 128 × 128. To enhance the strength and ability to handle variations in different datasets, our DL networks have incorporated data augmentation techniques (32, 33). These techniques involve random horizontal flipping, elastic transformations, and affine transformations.

**Table 1:** The data distribution of the three datasets for the training and test sets, including the number of normal and ADM retinas for each set.

**Dataset**	**Train**		**Test**		**Total**	
	**Normal**	**AMD**	**Normal**	**AMD**	**Normal**	**AMD**
DS1	23,794	7,214	1,237	434	25,031	7,648
DS2	1,006	530	291	147	1,297	677
DS3	17,320	3,480	2,268	243	19,588	3,723

**Figure 2:**
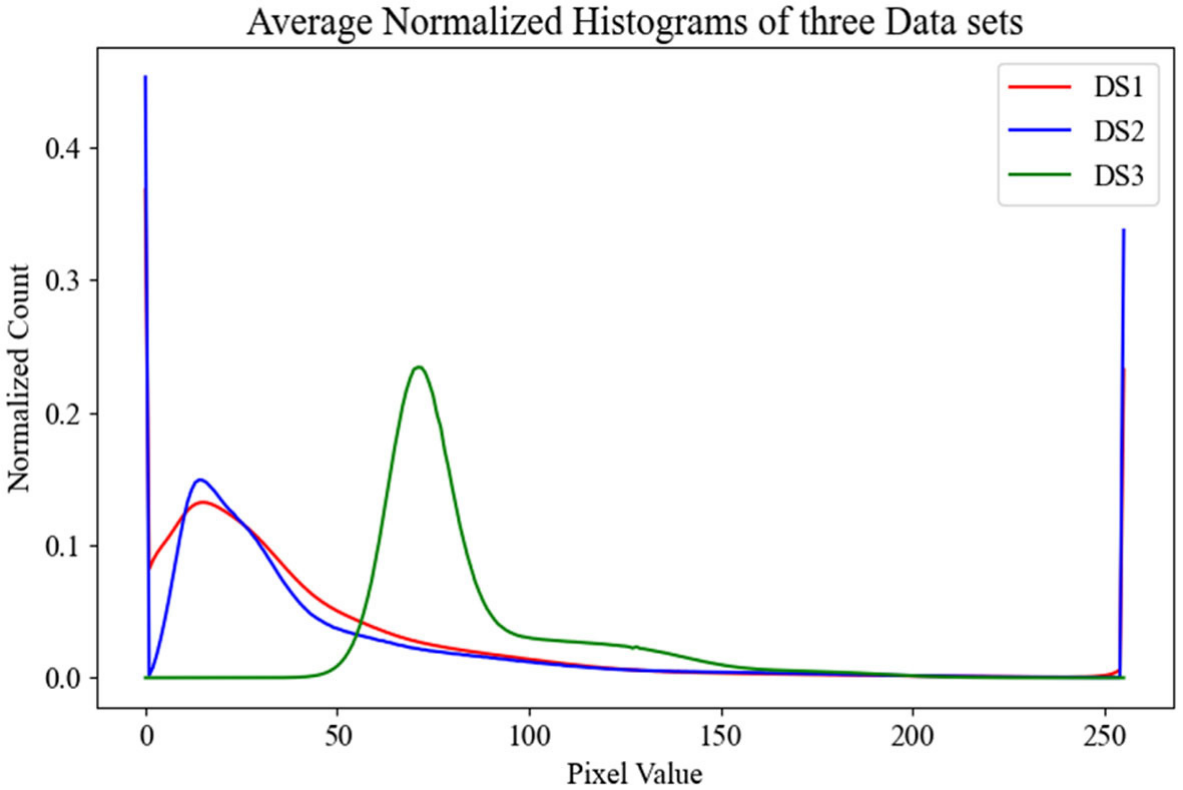
This figure displays the average normalized histograms for each dataset.

To gain insights into the distribution of our datasets, which in turn would aid in evaluating the performance of our models across the test sets, we calculated the average histogram of all OCT images in each dataset. These histograms are visualized in Figure 2, providing a clear picture of the individual dataset distributions. Upon observation, it is evident that the distributions of DS1 and DS2 are quite similar due to the fact that they both used the same device Heidelberg Engineering Spectralis OCT. In contrast, DS3 displays a wholly distinct distribution, likely stemming from the unique imaging protocols utilized in its creation.

### 2.2. Centralized and local models

**Figure 3:**
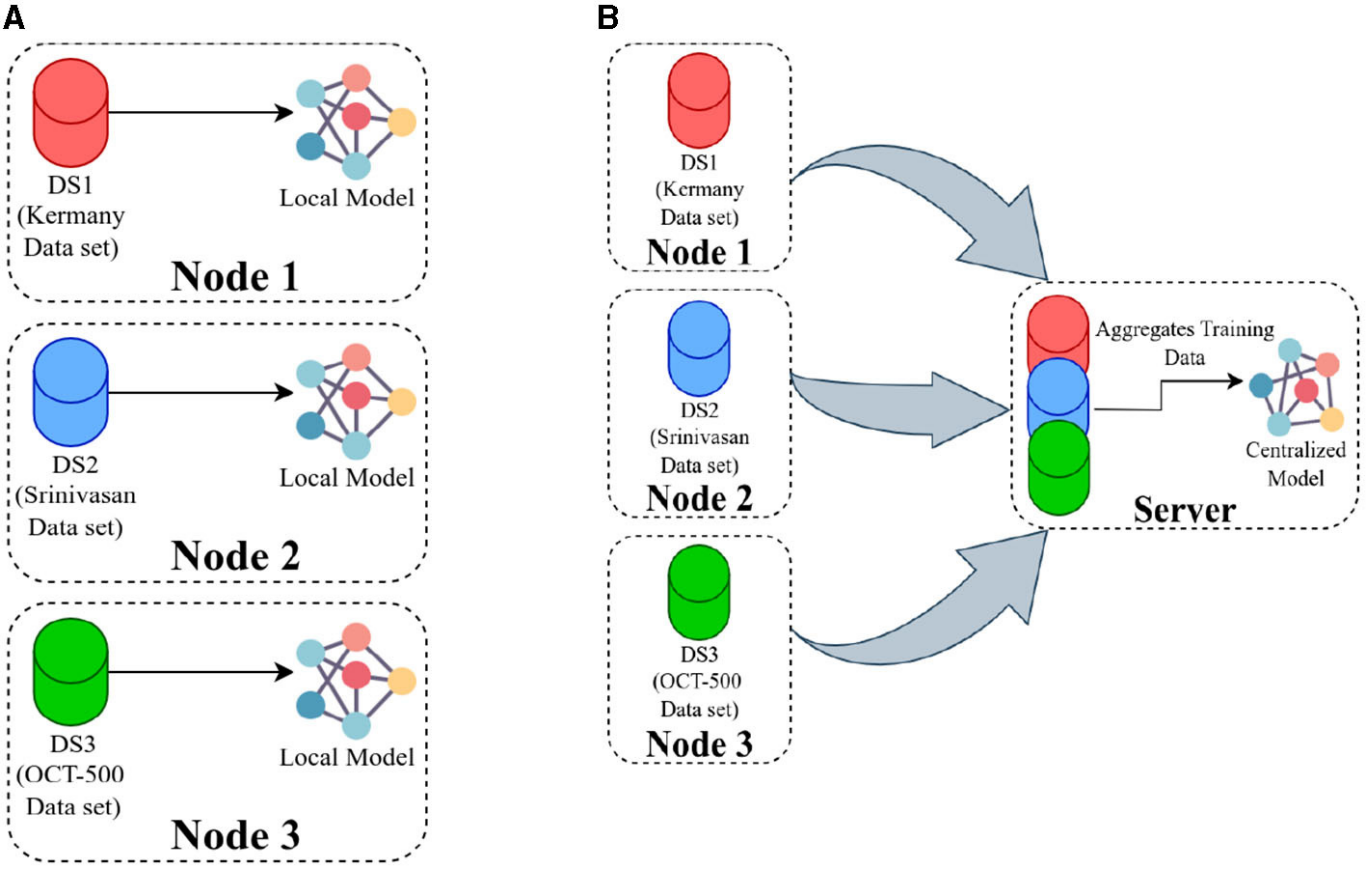
**(A)** Presents an overview of training local models, where each model is trained using data available at its respective location, and there is no communication between the nodes. In **(B)**, the server pools the data from all the nodes and then trains a single model using the combined dataset.

Having three datasets, each containing training and test sets, enabled us to train three separate models referred to as local models (Figure 3A). Concurrently, to establish a baseline comparison for the FL approach, we pooled all the data on the server and trained a model using the entire dataset, known as the centralized model (Figure 3B).

**Figure 4:**
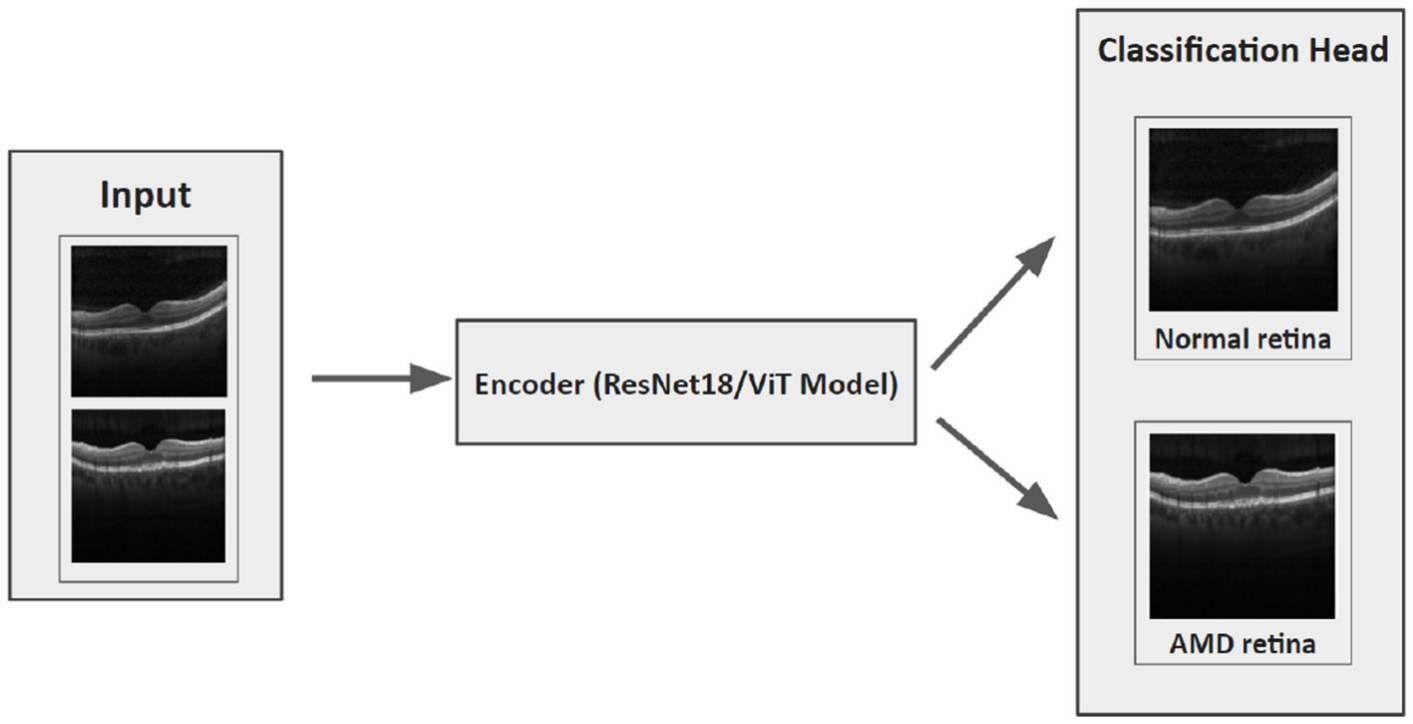
The architecture of our DL model.

Our hypothesis was that an FL model could be trained to deliver performance on par with a centralized model and it would surpass the performance of local models trained solely on locally available data. Then local and centralized models were subjected to performance assessment using all three test sets. This rigorous testing methodology provided us with a robust comparative analysis of the performance metrics of these models. The structure of each model is designed with two main components: an encoder and a classification head (Figure 4). After evaluating various options such as residual network (ResNet), vision transformers (ViT), VGG16, InceptionV3, and EfficientNet, we settled on ResNet18 with 11.2 million and ViT with 4.8 million parameters as the encoding mechanisms for our models, conducting thorough comparisons of their performances across diverse benchmarks. The ViT encoders consist of six transformer blocks and eight heads in the multi-head attention layer.

We utilized the Area Under the Receiver Operating Characteristics (ROC) Curve (AUC) as our metric for evaluation. Initial findings indicated that the Adaptive Momentum Estimation with Weight Decay (AdamW) surpassed the Stochastic Gradient Descent (SGD) in terms of performance at both the local and centralized levels, after hyperparameter optimization through grid search. The optimal hyperparameter combination was determined through the maximization of the AUC on the validation set, taking care to prevent data leakage from the test set. To examine the impact of the number of epochs (E) on the models, we trained the models with *E* = 10 and *E* = 100, implementing early stopping based on the AUC of the validation set and patience of ten epochs when *E* = 100. DS1 was processed using a computer (referred to as “node 1”) that was equipped with two Nvidia RTX A6000 graphics cards. DS2 was handled by a different computer (referred to as “node 2”). This machine was equipped with two Nvidia Titan V graphics cards. DS3 was processed on yet another computer setup (referred to as “node 3”). This particular machine had eight Nvidia GTX1080Ti graphics cards, which would be responsible for the computational demands of DS3. The summarized results can be found in Table 2.

**Table 2:** The AUC of the centralized and local models using E = 10 and E = 100 with early stopping on the validation AUC.

**Method**	**Test set**		
		**DS1**	**DS2**	**DS3**
**E = 10**			
Centralized (ResNet18)	92.78 ± 1.31	**98.9** **±** **0.64**	97.72 ± 1.5
Centralized (ViT)	85.64 ± 1.34	98.19 ± 0.59	**99.22** **±** **0.38**
Local DS1 (ResNet18)	**93.7** **±** **0.94**	98.83 ± 0.63	54.14 ± 2.68
Local DS1 (ViT)	85.23 ± 0.87	96.76 ± 0.66	88.08 ± 2.27
Local DS2 (ResNet18)	55.6 ± 1.86	80.4 ± 7.21	50 ± 0.0
Local DS2 (ViT)	51.07 ± 0.78	75.03 ± 3.96	53.63 ± 1.95
Local DS3 (ResNet18)	49.84 ± 0.56	54.65 ± 4.52	94.5 ± 2.67
Local DS3 (ViT)	48.39 ± 1.62	56.73 ± 6.53	86.3 ± 5.18
**E = 100**			
Centralized (ResNet18)	**94.58** **±** **0.62**	99.05 ± 0.4	98.91 ± 0.67
Centralized (ViT)	87.85 ± 1.2	**99.18** **±** **0.55**	**99.11** **±** **0.39**
Local DS1 (ResNet18)	93.97 ± 0.69	98.26 ± 0.87	51.74 ± 0.66
Local DS1 (ViT)	88.31 ± 1.5	97.7 ± 0.59	88.8 ± 2.74
Local DS2 (ResNet18)	57.03 ± 2.42	84.47 ± 8.1	50 ± 0
Local DS2 (ViT)	52.61 ± 1.39	83.16 ± 2.84	56.6 ± 3.18
Local DS3 (ResNet18)	49.99 ± 0.01	50 ± 0.0	91.98 ± 4.02
Local DS3 (ViT)	48.45 ± 2.83	55.49 ± 7.54	84.59 ± 4.19

Each dataset's best AUC value achieved by its corresponding model is highlighted in bold.

### 2.3. FL framework

Traditional FL algorithms involve a central server that oversees model updates and circulates the global model to all participating nodes. Local models are trained on the respective data and subsequently transmitted back to the server, where they are integrated into the global model (25). The primary FL algorithms used are FedAvg (23) and Federated Stochastic Gradient Descent (24), as well as their variations.

However, the decentralized character of FL introduces substantial challenges, especially in terms of data heterogeneity and distribution shifts. For instance, in ophthalmology, considerable variations in retinal images across different institutions can be attributable to factors such as the use of distinct imaging devices (34), heterogeneous patient populations (35), and inconsistencies in image acquisition protocols (36).

Addressing these challenges necessitates domain alignment, also referred to as DA. This essential process modifies an ML model trained on one domain to perform proficiently on a related domain. Numerous techniques have been proposed to mitigate the domain shift problem, making it crucial to implement these methods for successful DA. In our FL framework, we have compared four DA strategies alongside FedAvg: FedProx, FedSR, FedMRI, and APFL.

#### 2.3.1. FedProx

FedProx (37), is specifically designed to counter the data heterogeneity challenge in FL. It utilizes proximal regularization to incorporate a penalty term into the loss function and avoid overfitting. By maintaining local updates close to the initial global model parameters, FedProx is particularly useful when dealing with not non-independent and identically distributed data. This ensures each local model does not veer too far from the global model during training, yielding a more resilient global model that performs well across a broader spectrum of data distributions.

#### 2.3.2. FedSR

FedSR (38) simplifies the model's representation and encourages it to extract only essential information. This method employs two regularizers: an L-2 norm regularizer on the representation and conditional mutual information between the data and the representation given by the label. These regularizers limit the quantity of information the representation can contain. By enforcing these regularizers, FedSR facilitates learning data representations that generalize well across diverse domains, all while maintaining data privacy between nodes—a crucial advantage in an FL context.

#### 2.3.3. FedMRI

FedMRI (39) addresses the issue of domain shift that might surface during local node optimization. It does so through the implementation of a weighted contrastive regularization, which helps guide the update direction of the network parameters, thus directly rectifying any discrepancies between the local nodes and the server during optimization. This approach contrasts with traditional contrastive learning, which relies on identifying positive and negative pairs from data. In experiments involving multi-institutional data, FedMRI has demonstrated superior performance in image reconstruction tasks compared to state-of-the-art FL methods. As our task resided within the realm of binary image classification, we customized the FedMRI approach. Specifically, we excluded the decoder component and employed the weighted contrastive loss as an auxiliary loss exclusively.

#### 2.3.4. APFL

The goal of APFL (40) is to improve the overall performance of a model in an FL setup by considering the distinct data distribution of each participating node. This approach ensures data privacy and model customization. APFL achieves this by adding a level of personalization to the learning process. It involves learning a global model that every node shares, as well as a personalized model that caters to each node's unique data distribution. The global model identifies common patterns across all nodes, and the personalized model learns from node-specific patterns.

Our FL structure integrated three FL nodes with a central server, and it was developed based on the Flower framework (41). Before running the local training on these nodes, the server needed to be operational, necessitating the selection of a particular FL strategy, FL settings, and training configuration. The strategy oversaw several elements of the training and evaluation protocol, such as weight initialization and aggregation. FL settings outlined necessary parameters for FL training, encompassing the minimum number of FL nodes needed for training and subsequent evaluation. Further, the training configuration encapsulated requisite parameters for DL model training, including the number of epochs, learning rate, and weight decay.

**Figure 5:**
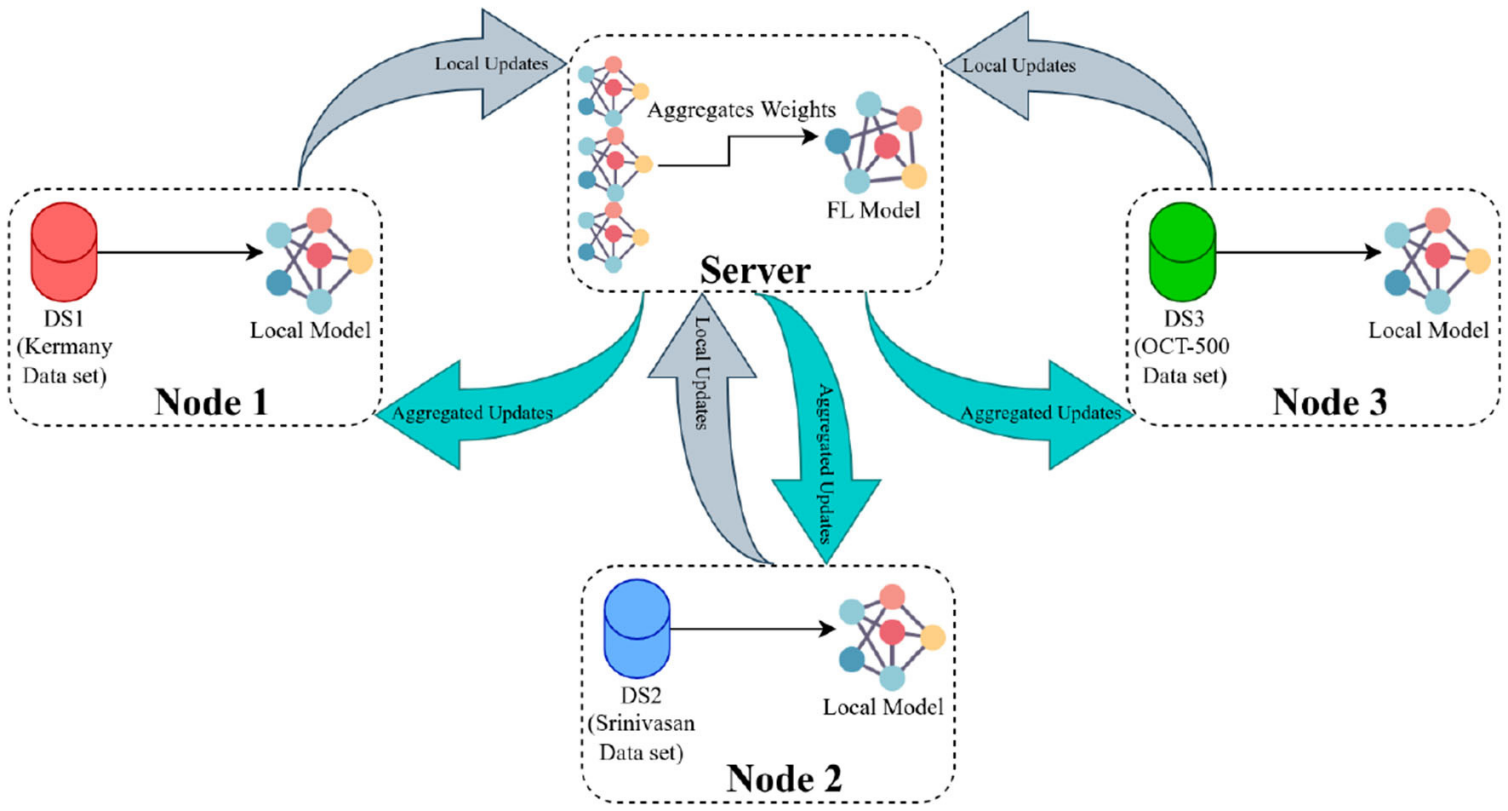
The FL framework operates in the following manner: The server initially distributes the primary weights to each participating node. These nodes then update their local models using the provided weights and initiate their training processes. Once training is concluded, the nodes relay their local weight updates (or local weights) back to the server. The server, in turn, computes the average of all submitted weights and circulates this consolidated update to all nodes as aggregated updates.

The procedure to train the FL model generally follows these steps (demonstrated in Figure 5): Initially, the FL strategy (options include FedAvg, FedSR, FedProx, FedMRI, and APFL) will be designated, as well as the FL settings such as the minimum number of FL nodes to start the training and evaluation, and training configurations (e.g., the number of epochs, learning rate, batch size, and weight decay). Subsequently, the server waits for the necessary minimum number of FL nodes to establish a connection. In our scenario, it needs exactly three nodes connected. Then the server dispatches the training configuration and the initial weights (based on the selected FL strategy) to each node. After receiving the weights from the server, each node updates its local model and starts the training process using the training configuration provided by the server. The training procedure primarily involves processing the local data through the model. The model's architecture can be viewed in Figure 4. Upon completion of the training, each node transmits its local model's weights back to the server. Finally, the server aggregates these weights using the designated strategy (such as FedAvg) and reciprocates by sending the updated weights back to each client, marking the conclusion of one round (*R*).

In this framework, there is an optional step called evaluation, where each local node assesses its performance after receiving the global FL model and evaluation configuration over local test sets. The evaluation configuration is similar to the training configuration and may contain various hyperparameters for model evaluation, such as batch size. After evaluation, the performance of each node is sent to the server to demonstrate the FL model's overall performance across all node test sets. Data heterogeneity can be handled by varying the number of local training epochs. This way, each round of training can be more productive, reducing convergence time and communication costs (42). To assess the training productivity of each strategy, we examined its AUC with three different allotted local training epochs per round in Table 3.

**Table 3:** The AUCs of the different FL methods on the three test sets with E = 1, E = 5, E = 10, and R = 10.

**Method**	**Test set**, ***R*** = 10		
		**DS1**	**DS2**	**DS3**
**E = 1**			
FedAvg (ResNet18)	90.47 ± 1.11	99.01 ± 0.27	54.26 ± 1.4
FedAvg (ViT)	82.4 ± 0.33	97.25 ± 0.19	93.95 ± 0.43
FedProx (ResNet18)	85.46 ± 2.11	91.75 ± 3.64	53.67 ± 3.02
FedProx (ViT)	82.4 ± 0.38	97.33 ± 0.23	94.03 ± 0.47
FedSR (ResNet18)	89.29 ± 1.27	**99.45** **±** **0.32**	59.12 ± 2.42
FedSR (ViT)	81.5 ± 0.24	95.01 ± 0.08	95.59 ± 0.58
FedMRI (ResNet18)	89.17 ± 0.99	95.46 ± 0.75	98.32 ± 0.33
FedMRI (ViT)	50 ± 0.0	50.2 ± 0.22	50 ± 0.0
APFL (ResNet18)	**90.94** **±** **2.23**	98.25 ± 0.52	97.91 ± 0.87
APFL (ViT)	83.35 ± 0.48	83.4 ± 2.8	**98.32** **±** **0.01**
**E = 5**			
FedAvg (ResNet18)	92.48 ± 0.74	99.42 ± 0.32	55.87 ± 4.28
FedAvg (ViT)	81.61 ± 9.39	98.47 ± 0.34	92.16 ± 9.4
FedProx (ResNet18)	92.11 ± 1.08	97.22 ± 1.1	65.1 ± 3.7
FedProx (ViT)	88.16 ± 0.36	98.32 ± 0.32	96.53 ± 0.45
FedSR (ResNet18)	92.99 ± 0.49	**99.79** **±** **0.16**	56.91 ± 3.6
FedSR (ViT)	85.58 ± 0.55	98.22 ± 0.38	97.44 ± 0.46
FedMRI (ResNet18)	91.9 ± 1.01	93.95 ± 3.57	98.01 ± 0.38
FedMRI (ViT)	73.09 ± 0.12	92.62 ± 0.11	86.31 ± 0.52
APFL (ResNet18)	**94.29** **±** **0.53**	99.59 ± 0.29	**98.96** **±** **0.43**
APFL (ViT)	86.95 ± 0.38	95.8 ± 0.69	97.55 ± 0.32
**E = 10**			
FedAvg (ResNet18)	89.88 ± 0.66	99.62 ± 0.13	54.54 ± 0.84
FedAvg (ViT)	88.55 ± 0.42	99.18 ± 0.26	98.07 ± 0.21
FedProx (ResNet18)	92.89 ± 0.63	99.54 ± 0.18	58.57 ± 4.27
FedProx (ViT)	88.86 ± 0.34	99.02 ± 0.23	98.17 ± 0.26
FedSR (ResNet18)	90.87 ± 0.68	**99.74** **±** **0.22**	54.07 ± 2.01
FedSR (ViT)	88.61 ± 0.45	99.12 ± 0.19	**98.23** **±** **0.3**
FedMRI (ResNet18)	93.16 ± 0.93	97.19 ± 0.96	97.48 ± 1.13
FedMRI (ViT)	81.84 ± 0.27	94.73 ± 0.43	94.09 ± 0.18
APFL (ResNet18)	**93.39** **±** **1.1**	99.23 ± 0.18	96.57 ± 1.94
APFL (ViT)	90.25 ± 0.47	93.91 ± 5.41	97.95 ± 0.2

The best AUC value achieved by each model for its respective dataset is highlighted in bold.

During each benchmarking session, one of the nodes played a dual role by serving as both a server and an FL node. The other two resources solely functioned as FL nodes and communicated with the server. Whenever *E* = 10, node 1 assumed the role of both server and FL node. When *E* = 5, node 2 became the server, and when *E* = 1, node 3 took on the role of server. The DL models at each local node were trained using the hyperparameters detailed in the preceding section. Note that, the value of *R* in all FL benchmarks is 10. The hyperparameters, input size, and image transformation have been applied as previously mentioned.

## 3. Results

The summary of outcomes from training a variety of local and centralized models is given in Table 2. These models are evaluated against three distinct test sets at the end of the training phase. The training process employed both ResNet18 and ViT encoders, and the table presents the corresponding performance metrics for each. In the latter part of Table 2, outcomes from training models at *E* = 100 are particularly highlighted. At *E* = 10, the local DS1 ResNet18 achieved superior performance on its native test set, while the centralized ResNet18 and ViT excelled on DS2 and DS3 test sets, respectively. With *E* = 100, centralized models topped the performance charts, with the ResNet18 encoder recording the highest accuracy rates of 94.58%±0.62 on DS1, and the ViT encoder reaching 98.18%±0.55 and 99.11%±0.39 on DS2 and DS3 test sets, respectively.

Moreover, FL strategies such as FedAvg, FedProx, FedSR, FedMRI, and APFL have been meticulously detailed in Table 3. These strategies have been examined in tandem with the employment of ResNet18 and ViT encoders, with the models being trained at *E* = 1, *E* = 5, and *E* = 10. To facilitate easier comprehension, the table specifically highlights in bold the highest AUC for each *E* value. Remarkably, a pattern emerges in the performance of the models on different test sets. The APFL ResNet18 performed the best for DS1. The FedSR ResNet18 showed superior performance for DS2. As for DS3, the APFL ViT, APFL ResNet18, and FedSR ViT performed the best at *E* = 1, *E* = 5, and *E* = 10 respectively. However, it is crucial to bear in mind that the optimal model should maintain a balanced performance across all test sets, and not merely excel in a single one. To ensure consistency, the parameter *R* has been maintained at a constant value of 10 throughout all the testing scenarios.

**Figure 6:**
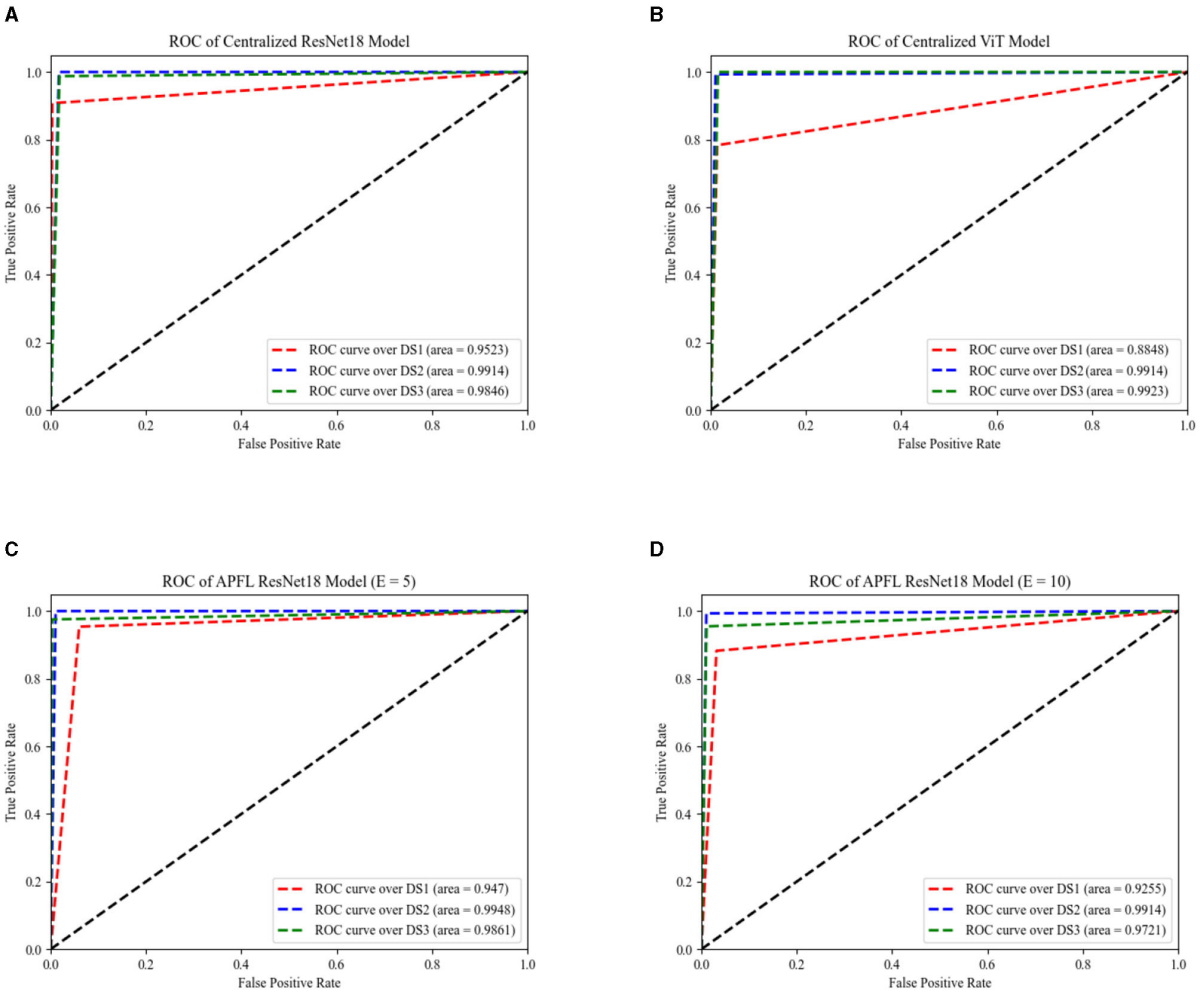
The AUC of centralized models across three datasets, as depicted by ResNet18 and ViT encoders, are illustrated in **(A, B)**, respectively. The same performance measurements, but this time using the APFL strategy with *E* = 5 and *E* = 10, are showcased in **(C, D)** correspondingly.

Figure 6 provides essential information on the performance of the centralized ResNet18 and ViT models across the three test sets at *E* = 100, with the patient parameter set to ten. It also features the exceptional performance of the APFL strategy, denoting it as the leading FL method in this problem.

**Figure 7:**
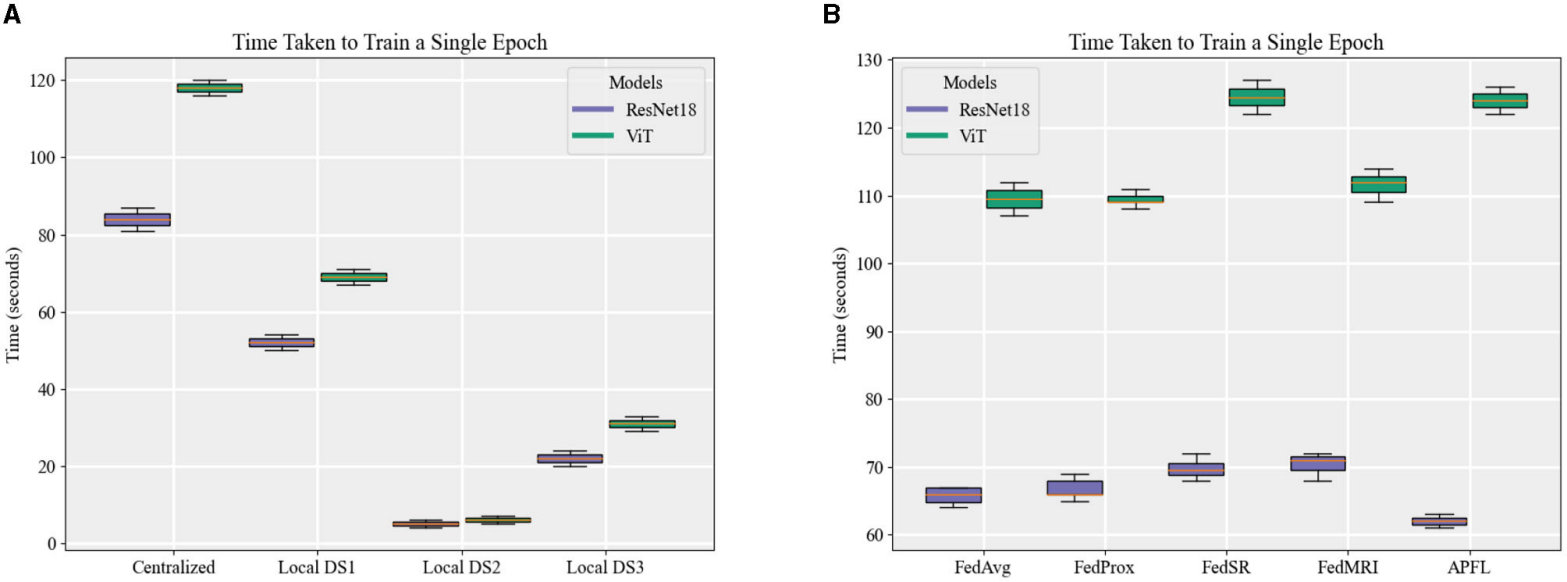
A comparison of the time taken to train an epoch for **(A)** local and centralized models, and **(B)** different FL strategies when local *E* = 1.

Figure 7 depicts the training duration for each model, with a noticeable pattern of longer training times for ViT models in comparison to the ResNet18 equivalents. This trend is consistently apparent across local, centralized, and FL models, even persisting through FL training iterations at *E* = 5 and *E* = 10. The time difference is minimal when training the local model using DS2—ResNet18 takes about 4–6 s, while ViT requires around 5–7 s. However, this difference grows when it comes to centralized and FL models, extending up to ~40 s for training one epoch. Keep in mind that the duration to train an FL model for one epoch is timed from the instant the server dispatches the initial weights to all nodes until it receives and aggregates all the parameters (FL training time). This calculation does not include the time spent on initializing the server, starting the nodes, connecting them to the server, and the evaluation stages. Due to this reason, FL strategies, with the exception of FedSR, tend to take less time to train than centralized models at *E* = 1. Notably, FedSR stands out as having the lengthiest training time among all the benchmarks.

## 4. Discussion

This study presented a comprehensive series of experiments exploring the comparative effectiveness of deploying DL models using local, centralized, and FL methodologies across three distinct datasets. The primary focus was the classification of OCT images into Normal and AMD binary categories, for which we utilized ResNet18 and ViT encoders. We also integrated four unique DA methods into our FL strategy to tackle the prevalent issue of domain shift. As our results show, DA FL strategies demonstrated impressive proficiency in training a global model well-suited to this specific problem, achieving competitive performance metrics in comparison to centralized ResNet18 and ViT models despite a lack of access to the entire dataset. These findings underscore the critical role of FL in healthcare settings, where data accessibility is often compromised due to feasibility issues and privacy concerns. By assuring patient confidentiality and facilitating significant insights from distributed learning, FL reinforces its importance in the future of healthcare analytics.

We opted for ResNet architectures given their documented proficiency in medical image classification tasks (43, 44). Their architectural depth facilitates intricate data pattern learning, and the availability of pre-trained models adds to their appeal (45). ViT was selected for its capacity to integrate global image context, a crucial attribute for enhancing medical image classification (46–48). Its architecture negates the need for task-specific designs, allowing intricate pattern recognition without specialized configurations.

Our experimental procedure for local and centralized DL models encompassed two distinct training scenarios: short-duration training over 10 epochs and extended training over 100 epochs. Our aim was to identify the model that, when trained over 100 epochs with equivalent training data, exhibited optimal performance, using validation AUC as the stopping criterion. We also examined the impact of varying the number of local epochs on the training efficiency of FL strategies, setting *E*-values at 1, 5, and 10.

Our research confirmed the expected superiority of centralized models over local ones, attributed to their unrestricted data access during training, especially when *E* is maximized at 100. A notable observation was the inconsistent performance of the local DS1 ResNet18 model across different test sets. While this model demonstrated commendable efficacy on its native and DS2 test sets, it faltered with DS3. This challenge arose from the brightness distribution disparity among DS1, DS2, and DS3, as visualized in Figure 2. Further analysis of the counterpart model, local DS1 ViT, emphasized the inherent strength of the ViT architecture's global feature focus, contributing to its notable performance (88.08%±3.17). However, the local DS3 and DS2 models displayed challenges in delivering high-quality results on tests outside their training environments. Factors like limited model generalization (for DS3) and inadequate training data (for DS2) might be responsible. Interestingly, local ResNet18 models outperformed their ViT counterparts on corresponding test sets. This likely results from the depth and parameter richness of the ResNet18 architecture, giving it an advantage over ViT models, since the capability of ViTs to decode intricate patterns amplifies with increased data volume (49).

Regarding FL training duration, one would theoretically expect parallel training (intrinsic to FL models) to be swifter than sequential training. Although we noted a minor reduction in training time for a single FL model epoch, the disparate dataset sizes (with DS1 being larger) hindered significant time gains over centralized models. Training disparities between nodes also introduced bottlenecks, with nodes 2 and 3 awaiting node 1's completion. This issue intensified as the *E*-value rose, leading to prolonged idle times for faster nodes. This phenomenon is exclusive to the training phase; during inference, all nodes utilize the same model, ensuring uniform inference times.

In the FL context, the performance of FedAvg, FedProx, and FedSR models, all utilizing a ResNet18 encoder, was found lacking on DS3's test set. This was unexpected, especially since FedProx and FedSR were crafted to counter domain shifts. This performance gap is rooted in data heterogeneity, which induces a drift in the learning trajectory. This drift, primarily aligned with DS1 and DS2, results in suboptimal outcomes when the aggregated FL model is tested on DS3. Interestingly, despite its modest performance on DS3, the FedSR ResNet18 model excelled across all *E*-values on DS2's test set. In contrast, the three strategies (FedAvg, FedProx, and FedSR) employing the ViT encoder, consistently achieved above 81% performance across all test sets. Given their inherent global feature focus, this comparison accentuates the potential advantages of using ViTs over ResNet18. The FedMRI strategy introduces a different dimension. FedMRI ResNet18 showcased promising results across all test sets, whereas its ViT counterpart struggled at *E* = 1 and was mediocre at *E* = 5. This underscores the necessity for refined hyperparameter tuning to determine the optimal weighting for FedMRI's contrastive loss when using ViT as an encoder. Lastly, the APFL strategy emerged as a standout FL approach, consistently delivering an AUC performance exceeding 83% across all tests, regardless of the encoder. Notably, the APFL ResNet18 model produced stellar results, often matching or even surpassing the performance of centralized models. For instance, on DS1's test set, the APFL ResNet18 model achieved an AUC score of 94.29%±0.53 at *E* = 5, closely following the 94.58%±0.62 achieved by the centralized ResNet18 model at *E* = 100. On DS2, the model reached a score of 99.59%±0.29, outperforming the centralized ViT's 99.18%±0.55 at *E* = 10. Similarly, on DS3's test set, this model showcased a competitive performance of 98.96%±0.43, slightly behind the centralized ViT model's score of 99.22%±0.38 at *E* = 10.

The success of the APFL approach can be attributed to its personalized layer, which tailors learning to node-specific data distributions, ensuring consistent and robust performance. This highlights the potential of FL models to compete with, and occasionally surpass, their centralized counterparts. As noted, the data was sourced from two distinct machines: Heidelberg Engineering Spectralist and RTVue-XR Optovue. Differences in imaging acquisition protocols led to variations in image brightness and texture, evident in image samples (Figure 1). Yet, APFL's personalization layer effectively addresses this by capturing and preserving the unique characteristics of each local node domain. Furthermore, APFL consistently outperforms prominent local models. In summary, our research contrasted the conventional FL strategy, FedAvG, with four domain adaptation strategies, utilizing two prevalent encoders: ResNet and ViT. It underscores the promise of FL strategies, particularly those incorporating adaptive personalization, in crafting robust models that yield consistent results across diverse datasets. This is particularly relevant in FL contexts where institutional data, like in DS2, is limited or where datasets, such as DS3, experience domain shifts. These strategies herald the development of top-tier models with enhanced generalization, vital for future projects emphasizing data privacy and decentralization.

However, our study is not without limitations. During the training phase, we opted for a relatively straightforward DL architecture and an aggregation policy rooted solely in a weighted average. Future endeavors will explore more intricate aggregation policies. Despite these constraints, our results provide invaluable insights into the comparative efficacy of simpler architectures for image classification tasks and enrich our understanding of FL strategies. We anticipate that delving into other FL strategies in subsequent research will further illuminate the nuances of these models' performance. The separate classification head also emerges as a potential area of focus, with intelligent weight aggregation policy and amplitude normalization potentially amplifying FL network efficiency (50). Lastly, investigating deeper models such as ResNet50, ResNet101, or ViTs with additional transformer blocks and more profound multi-layer perceptron architectures might shift performance dynamics and yield fresh insights.

## Data availability statement

Data and code are available at https://github.com/QIAIUNCC/FL_UNCC_QIAI. The original contributions presented in the study are included in the article, further inquiries can be directed to the corresponding author.

## Author contributions

SG: Conceptualization, Data curation, Formal analysis, Methodology, Software, Validation, Writing—original draft, Writing—review and editing, Investigation, Funding acquisition, Project administration, Resources, Visualization. JL: Writing—review and editing, Conceptualization, Formal analysis, Investigation. TL: Writing—review and editing, Conceptualization, Formal analysis, Investigation. SO: Writing—review and editing, Conceptualization, Formal analysis, Investigation. AT: Conceptualization, Data curation, Formal analysis, Funding acquisition, Investigation, Methodology, Project administration, Resources, Software, Supervision, Validation, Visualization, Writing—original draft, Writing—review and editing. MA: Conceptualization, Data curation, Formal analysis, Funding acquisition, Investigation, Methodology, Project administration, Resources, Software, Supervision, Validation, Visualization, Writing—original draft, Writing—review and editing.
